# Physical Activity Patterns and Incident Frailty in the National Health and Aging Trends Study

**DOI:** 10.1093/geroni/igaf122.643

**Published:** 2025-12-31

**Authors:** Anis Davoudi, Lacey Etzkorn, Sunan Gao, Brian Buta, Qian-Li Xue, Karen Bandeen-Roche, Amal Wanigatunga, Jennifer Schrack

**Affiliations:** Johns Hopkins University, Baltimore, Maryland, United States; Johns Hopkins Bloomberg School of Public Health, Baltimore, Maryland, United States; Johns Hopkins University, Baltimore, Maryland, United States; Johns Hopkins University, Baltimore, Maryland, United States; Johns Hopkins University, Baltimore, Maryland, United States; Johns Hopkins Center on Aging and Health, Baltimore, Maryland, United States; Johns Hopkins Bloomberg School of Public Health, Baltimore, Maryland, United States; Johns Hopkins Bloomberg School of Public Health, Baltimore, Maryland, United States

## Abstract

Frailty is characterized in part by slow walking speed and low self-reported physical activity (PA), but there is limited evidence linking objective amount, intensity, and patterns of daily PA to frailty incidence. A deeper understanding of the characteristics of PA most strongly associated with frailty may help illuminate new markers for prevention. We examined the association between wrist-worn accelerometry-derived patterns of daily PA and incident frailty in the National Health and Aging Trends Study (NHATS). Frailty was defined as having ≥3 criteria of self-reported exhaustion, unintentional weight loss, low self-reported physical activity, and low measured grip strength and slow walking speed; having 1-2 criteria was considered prefrail. Daily PA features included total daily activity volume (total counts, active minutes/day), intensity (most active 5-minutes (M5) counts/day), fragmentation(%), and variability (intra-daily variability(IV), inter-daily stability(IS)). Survey-weighted multivariable logistic regression models were used to study the association between each accelerometer feature and incident frailty 1-year later, adjusting for sociodemographics and clinical conditions. In 519 participants with ≥3 days of valid accelerometry data who were robust or prefrail at baseline (33.5% aged ≥80 years, 56% female), 13.6% became frail. Every 30/min higher baseline active time, 100,000 more activity counts/day, and 1000 more M5 counts/day were associated with 13%[95% CI: 4%-21%], 8%[1%-15%], and 35%[21%-46%] lower odds of incident frailty, respectively. Additionally, every 1% higher fragmentation and 0.1 higher IV were associated with 7%[4%-11%] and 18%[6%-33%] higher odds of frailty. Future studies should investigate using accelerometer measures of PA to predict frailty onset.

